# Transcriptional profiling of human bronchial epithelial cell BEAS-2B exposed to diesel and biomass ultrafine particles

**DOI:** 10.1186/s12864-018-4679-9

**Published:** 2018-04-27

**Authors:** Andrea Grilli, Rossella Bengalli, Eleonora Longhin, Laura Capasso, Maria Carla Proverbio, Mattia Forcato, Silvio Bicciato, Maurizio Gualtieri, Cristina Battaglia, Marina Camatini

**Affiliations:** 1 Department of Life Sciences, Center for Genome Research, University of Modena and Reggio Emilia, Via G. Campi 287, 41125 Modena, Italy; 2 PhD Program of Molecular and Translational Medicine, Department of Medical Biotechnology and Translational Medicine, University of Milan, 20090 Segrate, Italy; 3 Polaris Research Centre, Department of Earth and Environmental Sciences, University of Milano-Bicocca, Piazza della Scienza 1, 20126 Milan, Italy; 4 Department of Physiopathology and Transplantation, University of Milan, Via Fratelli Cervi 93, 20090 Segrate, Italy; 5 Unit of Environmental Chemistry and Interaction with Life (UCEIV, EA 4492), Université du Littoral Côte d’Opale 189A, Avenue Maurice Schumann, F-59140 Dunkerque, France; 6 Italian National Agency for New Technologies, Energy and Sustainable Economic Development – ENEA SSPT-MET-INAT, Via Martiri di Monte Sole 4, 40129 Bologna, Italy; 7 Department of Medical Biotechnology and Translational Medicine (BIOMETRA), University of Milan, Via Fratelli Cervi 93, 20090 Segrate, Italy; 8 Institute for Biomedical Technologies, National Research Council (ITB-CNR), Via Fratelli Cervi 93, 20090 Segrate, Italy

**Keywords:** Ultrafine particles, Biomass particles, Diesel particles, Lung disorders, BEAS-2B, RNA-seq, Time-course, Gene network, Environmental particles, Human health

## Abstract

**Background:**

Emissions from diesel vehicles and biomass burning are the principal sources of primary ultrafine particles (UFP). The exposure to UFP has been associated to cardiovascular and pulmonary diseases, including lung cancer. Although many aspects of the toxicology of ambient particulate matter (PM) have been unraveled, the molecular mechanisms activated in human cells by the exposure to UFP are still poorly understood. Here, we present an RNA-seq time-course experiment (five time point after single dose exposure) used to investigate the differential and temporal changes induced in the gene expression of human bronchial epithelial cells (BEAS-2B) by the exposure to UFP generated from diesel and biomass combustion. A combination of different bioinformatics tools (EdgeR, next-maSigPro and reactome FI app-Cytoscape and prioritization strategies) facilitated the analyses the temporal transcriptional pattern, functional gene set enrichment and gene networks related to cellular response to UFP particles.

**Results:**

The bioinformatics analysis of transcriptional data reveals that the two different UFP induce, since the earliest time points, different transcriptional dynamics resulting in the activation of specific genes. The functional enrichment of differentially expressed genes indicates that the exposure to diesel UFP induces the activation of genes involved in *TNFα signaling* via *NF-kB* and *inflammatory response*, and *hypoxia*. Conversely, the exposure to ultrafine particles from biomass determines less distinct modifications of the gene expression profiles. Diesel UFP exposure induces the secretion of biomarkers associated to inflammation (*CCXL2*, EPGN, *GREM1*, *IL1A*, *IL1B*, *IL6*, *IL24*, *EREG*, *VEGF*) and transcription factors (as *NFE2L2*, *MAFF*, *HES1*, *FOSL1*, *TGIF1*) relevant for cardiovascular and lung disease. By means of network reconstruction, four genes (*STAT3*, *HIF1*a, *NFKB*1, *KRAS*) have emerged as major regulators of transcriptional response of bronchial epithelial cells exposed to diesel exhaust.

**Conclusions:**

Overall, this work highlights modifications of the transcriptional landscape in human bronchial cells exposed to UFP and sheds new lights on possible mechanisms by means of which UFP acts as a carcinogen and harmful factor for human health.

**Electronic supplementary material:**

The online version of this article (10.1186/s12864-018-4679-9) contains supplementary material, which is available to authorized users.

## Background

Combustion processes are the principal sources of primary ultrafine particles (UFP) and emissions from diesel vehicles and biomass burning account for the majority of ambient UFP [1]. Exposure to these particles have been linked to cardiovascular and pulmonary diseases, including lung cancer [2, 3]. Epidemiological and clinical evidences exist for both acute and chronic effects of combustion particles. Long-term epidemiologic studies have reported an increased risk of mortality from cardiopulmonary symptoms and lung cancer [2]. Acute exposure to traffic particulate matter (PM) has been directly linked to triggering of myocardial infarction [3] and studies involving volunteers exposed to UFP highlighted a higher rate of adverse vascular effects [4, 5].

The mechanisms of PM and UFP toxicity are multiple and greatly depend on the different combustion conditions under which particles are generated and, ultimately, on their chemical composition [5]. Several in vitro and in vivo studies unraveled diverse aspects of PM toxicology. Biomass and diesel combustions emit primary nanoparticles and are among the most important contributors to the total emitted UFP [6]. While diesel particles have been largely investigated, less data are available for particles derived from biomass combustion. Recently, we investigated the physicochemical properties and associated cellular effects of diesel and biomass UFP produced under controlled laboratory conditions [7]. Here, we report the analysis of the transcriptional changes induced in human bronchial cells by the exposure to UFP produced by the diesel and biomass combustion.

Oxidative stress and inflammation are the main cellular responses driving the effects of air borne PM and combustion-derived [3, 8]. UFP from diesel exhaust and biomass burning are enriched in organic compounds, such as polycyclic aromatic hydrocarbons (PAHs) and their derived molecules, that bind to and activate the aryl hydrocarbon receptor (AhR) [9]. AhR is responsible for the transcription of genes involved in responses to DNA damage and in xenobiotic metabolism. Although other mechanisms may concur, the metabolism and consequent activation of PAHs by the xenobiotic pathway is considered the main mechanism of diesel-induced reactive oxygen species (ROS) generation and oxidative stress [1]. AhR has also been reported to directly participate in the activation of the inflammatory response by diesel and traffic-related PM [10, 11]. In addition, oxidative stress itself is considered a main mechanism of diesel-induced inflammation, as ROS formation may activate redox-sensitive transcription factors involved in the regulation of pro-inflammatory genes, such as *NF-κB* and *Nrf2* [8]. In mice, cardiovascular effects derived from the exposure to ambient and diesel PM have been related to airway inflammation and to the release of inflammation cytokines such as TNF-α and IL-6 [12–14]. Overall, these mechanisms might contribute to carcinogenesis, the complex process leading to tumor formation. DNA oxidative damage by ROS and DNA adducts formation by electrophilic reactive products of PAHs are known to be implicated in the initiation stage of carcinogenesis [2]. Both inflammation and oxidative stress can contribute to the promotion and progression of carcinogenesis by altering the expression of genes related to cell differentiation, growth, proliferation and migration. Inflammatory events also contribute to the modification of the tumor microenvironment, enhancing angiogenesis and suppressing the immune system [2, 15].

Although genome-wide approaches have been previously used to assess the effect of PM on health [16], the impact of UFP exposure on gene expression remains under-investigated leaving largely undefined the effect of UFP on the transcriptional dynamics in human cells. To elucidate how UFP from diesel vehicles and biomass burning emissions modulate gene expression dynamics in bronchial epithelial cells, we performed a time course RNA-seq experiment in BEAS-2B cells, after exposure to a single dose of these emissions. The design of time course experiment allowed identification of early transcriptional events, likely involved in the activation of pivotal signaling cascades, and of hallmark processes linking UFP exposure to molecular mechanism underlying human diseases.

## Methods

### Particle sample collection

Diesel particles were sampled from a Euro IV light duty vehicle without diesel particle filter (DPF), fuelled by commercial diesel and run over a chassis dyno. A “URBAN” Artemis Driving Cycle was used to represent the average “stop & go” driving conditions typical of a European city urban context. To collect the particles mass necessary for biological and chemical analyses, we performed 30 driving cycles. In particular, 5 cycles were performed at the beginning of the experimentation to set up the sampling condition and 5 at the end of the sampling campaign to confirm that the performances of the vehicle were not modified. The remaining 20 cycles were used to collect particles for the biological analysis and chemical characterization. To remove aggregates larger than 1 μm, particles were collected on Teflon filters (Whatman), using a DGI-1570 (Dekati Gravimetric Impactor, Finland). Biomass particles were produced by a modern automatic 25 kW boiler, propelled with prime quality spruce pellet. To improve volatile organic compounds condensation, particles were sampled on Teflon filters after dilution of flue gases with clean air. Filters were kept at − 20 °C immediately after sampling, until chemical characterization or UFP extraction for biological tests was performed. A chemical and morphological characterization of particles has been conducted as in [7]; briefly, transmission electron microscopy (TEM) analysis of both diesel and biomass samples showed aggregates of soot particles, with dimension lower than 50 nm. Chemical characterization showed that metal content was higher in diesel samples, with the exception of Mn and K that were higher in biomass. Diesel particles were characterized by the presence of transition metals such as Fe, Zn, Cr, Pb, V and Ni. Speciation showed a typical composition of PAHs associated with diesel soot with high levels of pyrene, phenanthrene, benzo[a]anthracene and dibenzo[a,h]anthracene, while the most abundant PAHs in biomass samples were fluoranthene and pyrene. The content of PAHs was higher in diesel (592 ng/mg) samples compared to biomass (55 ng/mg) ones. UFP exposed to cells were extracted from Teflon filters as described in [17]. Briefly, filters of the same source were pooled in a glass vial, covered with cold sterile water, and sonicated in an ice-cold water-bath (SONICA Soltec). Particle suspensions (Additional file 1: Figure S1) were dried into a desiccator weighed, and stored at − 20 °C until use. Particles were re-suspended in sterile water at a final concentration of 2 μg/μl just before use.

### Cell culture and experimental design of UFP exposure

Human bronchial epithelial cells BEAS-2B were maintained in LHC-9 medium at 37 °C and 5% of CO_2_. Cells for treatments were seeded at a concentration of 2.7 × 10^5^ cells/well in Cell BIND 6-well plates (Corning), and treated the day after with diesel or biomass UFP. Cell viability was assessed after 20 h of exposure to 2.5, 5, or 10 μg/cm^2^ (equivalent to 25, 50, and 100 μg/ml) of UFP by Alamar Blue assay accordingly to manufacturer’s instructions (Life technologies). Cells were sampled at 1, 4, 8, 16, and 20 h after a single exposure to 2.5 μg/cm^2^ (25 μg/ml) of UFP and compared to untreated controls at the same time of exposure.

### RNA extraction and RNA sequencing

BEAS-2B cells exposed to 2.5 μg/cm^2^ of UFP or medium alone were collected at 1, 4, 8, 16, and 20 h, lysed, and stored in QIAzol Lysis reagent (Qiagen, Hilden, Germany) until RNA extraction. Three independent biological replicates were examined at each time point in UFP exposed and paired controls samples for a total of 45 samples. Total RNA was extracted using the miRNeasy extraction kit (Qiagen, Hilden, Germany) and eluted in RNase free-water, according to the manufacturer’s recommended guidelines. Quality and quantity of the total RNA samples were evaluated with 2100 Bioanalyzer (Agilent, Santa Clara, CA) and Nanodrop 1000 (Thermo Fisher Scientific, Wilmington, DE), respectively. Only RNA samples with RIN score more than 9 were considered. Same RNA samples were used either for RNA-seq and for qPCR validation. RNA-seq library was prepared from 1.5 μg of total RNA using TruSeq Stranded mRNA Library Prep Kit in paired end format (Illumina) following the manufacturer’s recommendations. RNA sequencing was carried out on Illumina HiSeq2500 platform (Illumina) at IGA technology service (Istituto Genomica Applicata, Udine, Italy). RNA-seq raw and processed data are deposited in the Array Express archive under the accession number E-MTAB-5157.

### Bioinformatics analysis

Read quality assessment and *trimming* were obtained by means of fastQC (version 0.11.3) and Trimmomatic (v. 0.33) [18], respectively. First 15 bases from the start of the reads were removed by the *headcrop* function of Trimmomatic tool. Then, raw reads were aligned to the human genome version hg19 with TopHat [19] (version 2.1.0) changing only the mate inner distance and its S.D. to 250 and 300, respectively, respect to default parameters. Read counts for UCSC annotated genes were calculated using the *htseq-count* function of HTSeq tool [20] (version 0.6.0) with the *mode* option set as *union* (default) for ambiguously-mapped reads.. Normalization was carried out with *edgeR* package [21] (version 2.12.0,) in R-3.2.2. Raw counts were normalized according to library size to obtain counts per millions (cpm) and only genes with a cpm ≥1 in at least three samples were retained for subsequent analyses.

Differentially expressed genes (DEGs) at each time point between treated and untreated samples were identified using a paired design with the likelihood ratio tests of the *glmLRT* function in *edgeR* package and considered significant if the *p*-value was ≤0.05 and the absolute Fold Change (FC) ≥1.2. DEGs were annotated with the ToppGene suite (https://toppgene.cchmc.org/) [22] and prioritized by literature mining using PubMatrix [23].

Enrichment analysis was performed using the pre-ranked tool of Gene Set Enrichment Analysis [24] (GSEA, version 2.2.1,) and the gene sets of the Molecular Signature Database Hallmarks collection (i.e., 50 gene sets representing well-defined biological processes; MSigDB http://software.broadinstitute.org/gsea/msigdb). Specifically, genes were ranked separately for each time point using the fold change value between treated and untreated samples. Gene sets smaller than 15 or larger than 500 genes were excluded; gene sets passing this filter were considered significant if the False Discovery Rate (FDR) was ≤0.05 when using 1000 permutations of gene sets. To test DEGs enrichment for diseases signatures we used the ToppGene tool (https://toppgene.cchmc.org/) with the DisGeNET database (http://www.disgenet.org/), considering significant only the diseases with FDR corrected *p*-value ≤0.05.

Groups of genes with a differential temporal expression profile in exposed cells (as compared to controls) were identified using the *next-maSigPro* method of the *maSigPro* [25, 26] R package (version 1.34.1). Briefly, the *p.vector* function was used to compute a regression fit for each gene in both matrixes of cpm counts for BEAS-2S exposed to diesel and biomass UFP separately. Temporally differentially expressed genes were detected using the generalized linear model (glm) setting a p-value ≤0.05 after FDR correction. In the glm, the *family* parameter was set to *negative.binomial* to adapt the theoretical distribution of the read counts, the *counts* parameter was set to true, and the *theta* parameter which corresponds to the dispersion of the distribution_ was left to the default value of 10, since any variation (e.g., increasing to *theta* = 20 or decreasing to *theta* = 5) induced no evident changes in the number of DEGs. Genes with significant expression changes over time were detected with a second regression model by the *t.fit* function, selecting the *two.ways.backward* procedure for the regression method and *theta* = 10 for the negative binomial distribution. The final selection of temporally differentially expressed genes was obtained filtering the result of the second regression model with the *get.siggenes* function, with the *R-squared* parameter set to 0.7 and the *vars* parameter to *groups.* Finally, significant genes have been grouped into k = 9 groups (default value) with the *see.genes* function, using *hierarchical* and *two.ways.backward* as clustering method and regression procedure, respectively. Genes used in the network reconstruction were selected among those satisfying at least one of the following criteria: i) a significant modulation (DEGs) in at least one time point; ii) the inclusion in at least one time point in the core enrichment of 4 manually selected significant gene sets from GSEA analysis (*TNF-α signaling* via *NF-κB*; *inflammatory response*, *epithelial mesenchymal transition(EMT)*; and *xenobiotic metabolism)*; iii) a PubMatrix total score higher or equal to 15, corresponding to at least 15 publications containing both the gene name and the terms *cardiovascular diseases*, *lung cancer*, or *lung diseases*. The gene network was created in Cytoscape [27] (version 3.3) using the Reactome FI plugin [28] (version 5.0). For diesel UFP, 129 genes selected using the above mentioned criteria were inputted as a list to the Reactome FI, which reconstructed gene-gene interactions using the pathway-based Reactome Functional Interaction (FI) database (version 2015). The Reactome Functional Interaction (FI) database contains both manually annotated and predicted gene-gene relationships [29]. Since genes modulation could have been induced at protein level only, we used the *linker genes* option to add putative linker genes in the network. Linker genes are automatically selected by the plugin algorithm among the genes that connect the higher number of genes of the input list according to the Reactome FI database annotation. Moreover, we discarded single genes, i.e. genes not linked to any node of the network (*n* = 7). Finally, fold changes have been visualized in the network using the pie chart style of the Cytoscape EnhancedGraphics plugin [30] (version 1.0.3).

### Validation of selected genes and proteins modulated by diesel exposure

Expression levels of selected genes were validated by qPCR using a customized RT^2^ PCR profiler system (Qiagen, Hilden, Germany) and the TaqMan technology. The qPCR validations were carried out on same RNA samples used for RNA-seq analysis, comprised 4 time points (4, 8,16, and 20 h) and 3 biological replicates of BEAS-2B exposed to diesel UFP. The expression of each gene within each sample was normalized using the average expression levels of three housekeeping genes (*ACTB*, *B2M*, *GAPDH*). For *IL1B* gene, we used a pre-designed TaqMan® Gene Expression assay (Hs01555410_m1) and TaqMan® Universal Master Mix (Applied Biosystem, Foster City, CA) using *ACTB* genes (Hs99999903_m1) as reference. All qPCR reactions were processed on an Applied Biosystem 7900HT real time PCR machine. The fold change was calculated for each condition using the 2^-∆∆Ct^ method comparing ∆Ct of UFP treated cells to ∆Ct of control untreated cells.

To validate results at the protein level, the expression of four secreted proteins (i.e., IL-6, VEGF, EREG, and IL-24) has been quantified with the ELISA assay (IL-6 and VEGF: Life Technologies; EREG and IL-24: Elabscience) according to the manufacturer’s guidelines. The absorbance of each sample was measured using a Multiplate Reader Ascent (Thermo Fisher Scientific) at 450 nm and 630 nm and data analyzed with Ascent Software. Data are reported in pg/ml as mean and standard error of mean (SEM) of three independent experiments. Statistical analyses were performed in Sigma Stat 3.1, using one-way ANOVA with Dunnett’s or Dunn’s post hoc test and *p*-value< 0.05 for statistical significance.

## Results

To investigate the effects of UFP on the gene expression dynamics, we analyzed the transcriptional profiles of BEAS-2B cells exposed for 1, 4, 8, 16, and 20 h to a single dose of 2.5 μg/cm^2^ (corresponding to 25 μg/ml) of UFP of diesel and biomass combustion. We designed the time course in order to monitor transcriptional changes arising immediately after the exposure (e.g., 1 h) and during a period that could represent a fair approximation of the exposure in a real setting (i.e., 20 h). To determine the UFP dose for the time course experiment, first we assessed cell viability at various doses (25, 50, and 100 μg/ml) using the Alamar Blue assay (Additional file 2: Figure S2) and then we monitored by qPCR the transcription of few selected genes known to be involved in the cellular response to UFP exposure; finally, we selected the lowest (non-cytotoxic) dose with an effect on gene modulation (i.e., 25 μg/ml). We quantified gene expression levels using RNA sequencing. The overall quality of reads from RNA-seq was good: the quality metrics only indicated the presence of a general bias in the per-base sequence content for the first 12–15 bp (the difference between A and T, or G and C was greater than 20% in the first 15 bases)*,* most likely due to an unbalanced selection of random primers. Furthermore, to elevate the mapping quality, we trimmed out the first 15 bases from all reads prior to alignment. On average, we obtained 10 million mapped reads with a mapping rate of 90.1% in the 45 analyzed samples (Additional file 3: Table S1). Starting from a total of 25,369 UCSC annotated human genes, after quantification and normalization, we retained and considered for the subsequent analyses 13,309 genes with a transcriptional signal greater than 1 count per million (cpm) in at least 3 samples (Additional file 4: Figure S3).

### Exposure to diesel and biomass UFP induces specific transcriptional patterns in BEAS-2B cells

**Fig. 1:**
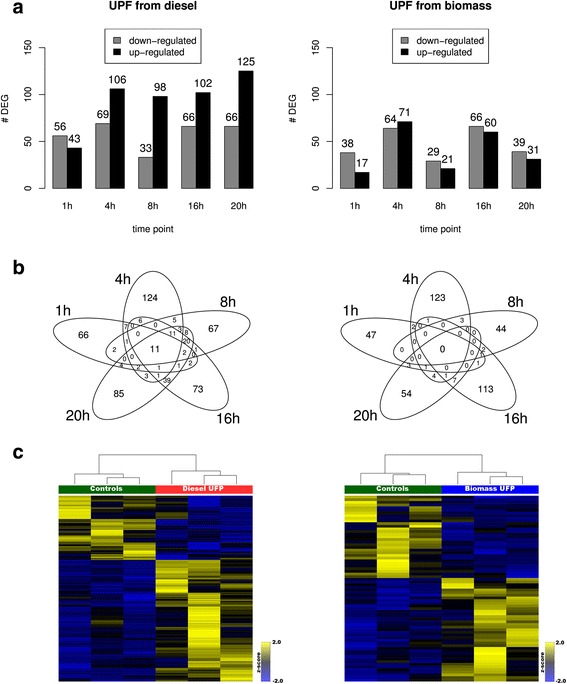
Analysis of the transcriptional differences of BEAS-2B cells exposed to diesel (left panels) and biomass UFP (right panels) as compared to controls. **a** Number of differentially up- and down-regulated genes at each time point. **b** Overlap of DEGs identified at each time point. **c** Hierarchical clustering of the 191 and 70 genes differentially expressed after 20 h of exposure to diesel and biomass UFP, respectively

Differential expression analysis with *edgeR* paired design identified a variable number of differentially expressed genes (DEGs) across the five time points in samples exposed to diesel and biomass UFP as compared to un-exposed controls (Fig. 1a and Additional file 5: Tables S2 and Additional file 6: Table S3). Globally, the exposure to diesel resulted in a higher number of modulated genes (*n* = 545) with respect to biomass (*n* = 407) at any time point (Fig. 1a). Interestingly, the number of DEGs induced by exposure to diesel UFP increased with time whereas the magnitude of the transcriptional modulation was variable across the time course for biomass*.* In general, the exposure to diesel UFP determined larger variations of the expression levels as compared to biomass. Overall, diesel and biomass induced the transcriptional modulation of 94 common genes (Additional file 7: Table S4), the majority of which (71) displayed the same pattern of expression along the time course. Among them, we found genes involved in *translation* (*RPL27, RPL36, GSPT2, RPLP2, RPS12, RPS15A*) and *AhR* (*CYP1A1, CYP1B1, AHRR*) pathways and genes encoding soluble factors involved in inflammation process and signaling (*IL24, IL15, TNFSF9, NTF4, EPGN, IL1B*). Few DEGs displayed a differential expression with respect to controls in more than one time point within the same treatment and only the exposure to UFP from diesel induced the statistically significant up-regulation of 11 genes along the entire time course (Fig. 1b). In particular, five of these (i.e., *CYP1A1, CYP1B1, IL24, ADAMTS15,* and *SHISA2*) showed an almost constantly growing up-regulation along the entire experiment, while six genes (*EPGN, IL1A, IL1B, IGFBP1, TIPARP,* and *NPTX1*) displayed a variable up-regulation at the different time points (Additional file 8: Figure S4). Despite some small differences in the three biological replicates, expression data of DEGs were able to clearly partition exposed cells from controls (Fig. 1c). To validate relevance of identified signatures in lung diseases, we compared DEGs to publicly available datasets. We limited the analysis to genes most constantly modulated during time, e.g. in at least 3 out of 5 time points, for a total of 53 and 3 genes for diesel and biomass, respectively. By ToppGene enrichment analysis with the DisGeNET database, we observed a significant involvement to several cancer-related disorders for DEGs induced by diesel UFP: *Squamous Cell Carcinoma* (FDR *p*-value: 4.791E-7), *Chemical Carcinogenesis* (FDR p-value: 6.405E-5), *Squamous cell carcinoma of the head and neck* (FDR p-value: 6.405E-5) and *Lung Adenocarcinoma* (FDR p-value: 8.653E-5) were among top significantly enriched diseases. No significant diseases were found for biomass UFP-induced DEGs because of the low number of genes.

**Fig. 2:**
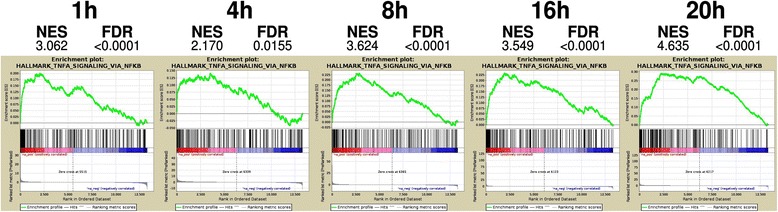
Enrichment plots along the entire time course of the *TNF-α signaling* via *NF-kB* hallmark gene set in BEAS-2B cells exposed to diesel UFP. Enrichment plots show an overrepresentation of genes on the left of the graph (vertical black lines), corresponding to a significant enrichment in up-regulated genes of this gene set after exposure to diesel UFP. The green line indicates the trend of the enrichment score (ES) with genes mostly contributing to the gene set enrichment located on the left with respect to the ES maximum

To functionally characterize transcriptional patterns, we applied the pre-ranked gene set enrichment analysis (GSEA) to 50 hallmark gene sets, i.e., to lists of genes summarizing well-characterized biological mechanisms with coherent expression levels. GSEA highlighted that exposure to diesel and biomass UFP induced the modulation of 42 and 33 hallmark gene sets in at least 1 time point, respectively (Additional file 9: Table S5 and Additional file 10: Table S6). The more massive modulation occurred at 20 h with 33 gene sets significantly modulated by diesel and 20 by biomass exposure (with a 95% statistical confidence, FDR ≤ 0.05). *TNF-α signaling* via *NF-κB, Hypoxia* and *Inflammatory response* gene sets resulted up-regulated by both treatments after 20 h of exposure (Table 1). In cells exposed to diesel UFP, the gene set of *TNF-α signaling* via *NF-kB* was strongly and steadily enriched across the entire time course differently from other gene sets (as *P53 pathway*, *EMT*, and *xenobiotic metabolism*) that were mostly enriched only after 20 h of exposure (Fig. 2).

**Table 1:** Top15 enriched gene sets in BEAS-2B cells after 20 h of exposure to UFP from diesel and biomass (FDR ≤ 0.05). Gene sets are from the of the MSigDB hallmarks collection; size is the number of genes in the gene set and NES, the normalized enrichment score, accounts for the overrepresentation of a gene set at the top or bottom of a ranked list of genes. Positive NES indicates enrichment in treated samples, instead negative NES in controls

Gene set	Size	NES
Enriched in BEAS-2B cells exposed to diesel UFP		
TNFA_SIGNALING_VIA_NFKB	182	4.635
P53_PATHWAY	185	3.383
HYPOXIA	168	3.145
EPITHELIAL_MESENCHYMAL_TRANSITION	173	3.063
XENOBIOTIC_METABOLISM	141	2.814
COAGULATION	80	2.723
ESTROGEN_RESPONSE_EARLY	166	2.645
CHOLESTEROL_HOMEOSTASIS	70	2.411
REACTIVE_OXIGEN_SPECIES_PATHWAY	43	2.389
GLYCOLYSIS	174	2.318
INFLAMMATORY_RESPONSE	127	2.272
APICAL_JUNCTION	150	2.270
G2M_CHECKPOINT	197	−2.819
PROTEIN_SECRETION	92	−3.075
E2F_TARGETS	199	−3.502
Enriched in BEAS-2B cells exposed to biomass UFP		
HYPOXIA	168	2.920
MTORC1_SIGNALING	194	2.555
PROTEIN_SECRETION	92	2.534
TNFA_SIGNALING_VIA_NFKB	182	2.363
FATTY_ACID_METABOLISM	130	2.350
ANDROGEN_RESPONSE	89	2.337
G2M_CHECKPOINT	197	2.335
E2F_TARGETS	199	2.330
INTERFERON_GAMMA_RESPONSE	158	2.263
MYC_TARGETS_V1	198	2.222
INFLAMMATORY_RESPONSE	127	2.115
BILE_ACID_METABOLISM	76	2.080
COMPLEMENT	139	2.049
TGF_BETA_SIGNALING	50	1.875
MYOGENESIS	123	−2.445

Interestingly, when considering genes mostly contributing to the enrichment of the *TNF-α signaling* via *NF-kB* gene set at the various time points, we identified 21 core enrichment genes (i.e., *AREG, CDKN1A, CXCL2, EGR1, F3, FOSL1, ICOSLG, IER3, IL1A, IL1B, IRF1, LIF, MAFF, NFE2L2, PHLDA1, SERPINB2, SLC16A6, TGIF1, TIPARP, TSC22D1, VEGFA*) conserved across all time points. Some of these genes code for secreted proteins (as *IL1A, IL1B, LIF, EREG, CXCL2, F3, VEGFA*) and transcription factors or modulators (as *NFE2L2*, *MAFF*, *HES1*, *FOSL1*, *TGIF1*) and their expression resulted significantly modulated in at least 1 time point (Additional file 7: Table S4). In general, gene expression data from cells exposed to biomass UFP returned lower normalized enrichment scores (NES) and smaller number of DEGs in the core enrichment of modulated pathways indicating a milder modulation of hallmark gene sets by biomass as compared to diesel. For instance, none of the genes comprised in the core enrichment of the hypoxia pathway, the most enriched gene set after exposure to biomass UFP (Table 1), resulted significantly differentially expressed, suggesting a uniform but modest modulation induced by biomass UFP.

#### Exposure to diesel and biomass UFP modulates transcriptional dynamics in BEAS-2B cells

**Fig. 3:**
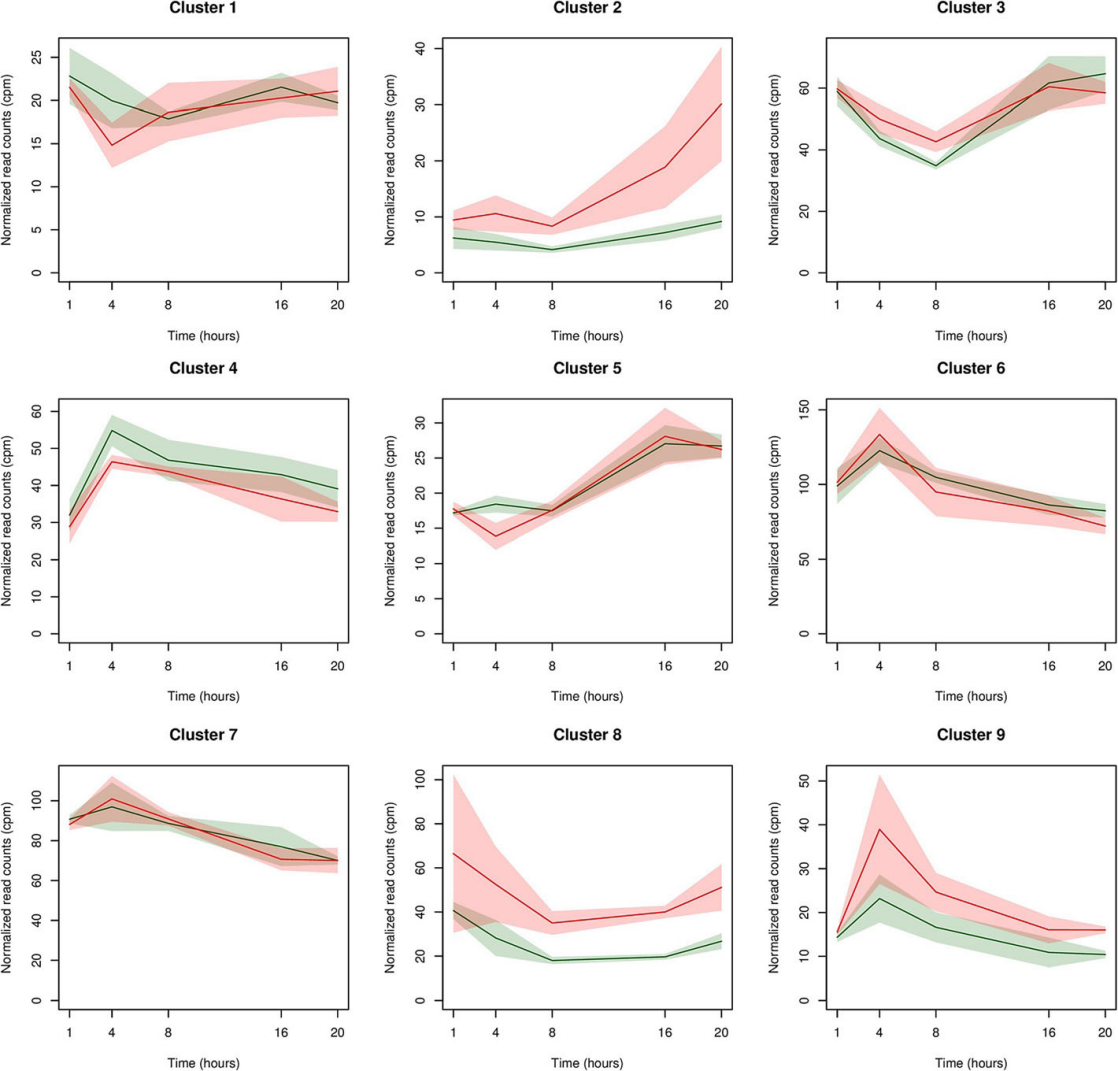
Expression profiles of genes showing statistically significant expression changes over time in BEAS-2B cells exposed to diesel UFP. Genes have been grouped into 9 clusters showing distinct expression profiles during the time of the experiment. For each plot, the expression values of the clustered genes are represented in either control cell lines (green) or cells after UPF diesel exposure (red), respectively. Solid line indicates the median instead shadow the median ± S.D. (standard deviation) of the expression values at each time point

To capture variations in the transcriptional dynamics after exposure to diesel and biomass UFP, we identified genes showing statistically significant expression changes over time using the *next-maSigPro* method of the *maSigPro* R package [24, 25]. *maSigPro* revealed profound temporal changes in the expression of 120 and 96 genes in BEAS-2B cells exposed to diesel and biomass UFP, respectively, at a 95% confidence level (Additional file 11: Table S7). Using hierarchical clustering and *maSigPro* default parameters, significant genes were grouped into nine clusters showing distinct expression profiles during the time of the experiment (Fig. 3 and Additional file 12: Figure S5 and Additional file 11: Table S7). Overall, we observed that exposure to diesel UFP exerted a stronger effect on the modulation of transcriptional dynamics in comparison with biomass UFP. Indeed, upon diesel exposure, four groups of genes (i.e., those comprised in clusters 4, 6, 7, and 9) showed a peak of expression at early time points (4 h–8 h); two clusters displayed an increasing trend over time after 8 h of exposure (clusters 2 and 5), while genes in cluster 3 and 8 had a sharp decrease of their expression level at 8 h (Fig. 3). In particular, cluster 9 presents some of the most up-regulated genes (as *EPGN, GREM1, HMOX1, SERPINB2*), cluster 2 comprises *AHRR*, *CYP1A1*, *CXCL2, IRAK2, TNFAIP3*, *IL24*, *IL6*, and *VEGFA*, while cluster 8 includes *CYP1B1, MAFF, LIF,* and *TIPARP* (Fig. 3). Upon exposure to biomass UFP, three groups of genes (clusters 1, 4, and 5) showed a peak of expression at early time points (4 h–8 h), while genes in clusters 7 and 9 increased their expression during the entire time course (Additional file 12: Figure S5). Annotation analysis of the gene groups highlighted that exposure to diesel UFP activated biological processes such as response to *oxidative stress* and *blood vessels development* while UFP from biomass exposure induced a significant enrichment in genes belonging to *gene expression* (*data not shown*).

### Reconstruction of the gene networks in BEAS-2B cells exposed to diesel and biomass UFP

To extract regulatory relations we applied a gene network analysis to the gene expression data of BEAS-2B exposed to UFP. To build the regulatory networks we selected genes that were differentially expressed or present in the core enrichment of significantly enriched gene sets in at least one time point (Additional file 13: Table S8). The application of these criteria led to the reconstruction of the regulatory network only for cells exposed to UFP derived from diesel, while the low number of DEGs and of genes in the core enrichment of GSEA hampered the reconstruction of a network based on the transcriptional profile of cells exposed to biomass UFP. For cells exposed to diesel UFP, the network comprised 164 genes and 576 interactions, mainly representing “*expression regulation”, “activation*” and “*phosphorylation*” types of regulation (Additional file 14: Table S9). The network architecture highlighted the presence of several interconnected sub-networks including several genes (nodes) interacting with many DEGs (Additional file 15: Figure S6).

**Fig. 4:**
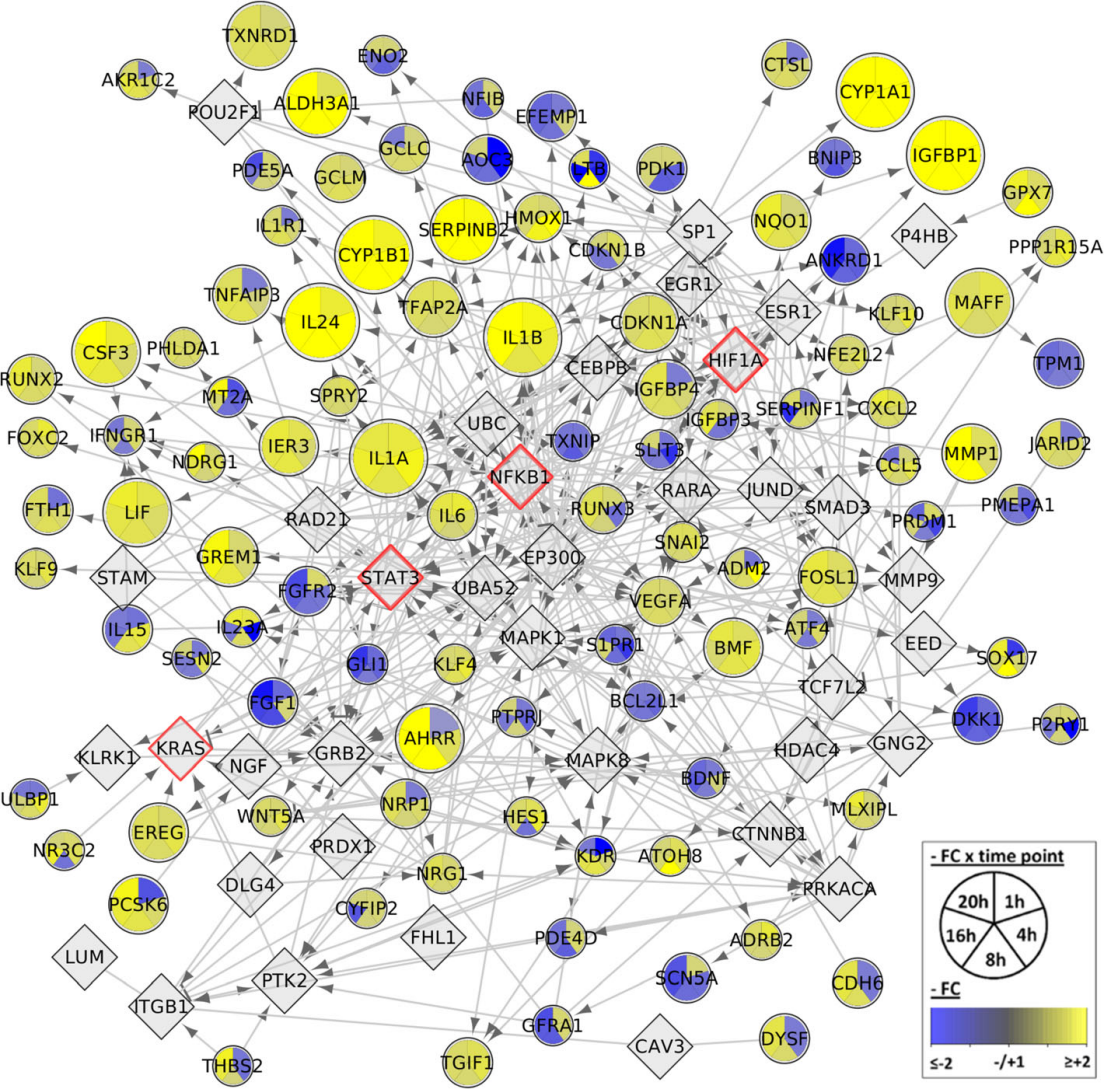
Network analysis of selected DEGs in cells exposed to from diesel UFP. The initial 545 DEGs were filtered by the presence in the core enrichment of four significant hallmarks GSEA (*TNF-α signaling* via *NF-κB*, *Inflammatory response*, *EMT*, *Xenobiotic metabolism*) or by literature mining prioritization; (further details on DEGs selection are in section Material and Methods, and in Additional file 14: Table S9). Of the 129 resulting DEGs, network analysis identified a biological interaction for 122 genes (rounded shape) and 42 further “linker” genes (diamond shape). Some of the most interesting linker genes are investigated in the Discussion section (red board). Grey lines indicate manually curated interactions according to Reactome FI database. Size of DEGs nodes is directly proportional to the number of differentially expressed time points; the FC at each time point in cells exposed to UFP from diesel vs control is also indicated. The complete network inclusive of the predicted interactions is available as Additional file 15: Figure S6

Overall, we identified 42 node genes that, although not differentially expressed, displayed direct interactions with either up or down-modulated genes (Fig. 4). By literature mining we identified some interesting genes (as *STAT3*, *HIF1*a, *NFKB*1, *KRAS*) that have been reported to play an important role in biological processes as *inflammation*, *EMT*, *coagulation* and *angiogenesis*, which are at the basis for cancer and cardiovascular diseases. Overall, the gene network allowed discovering hidden regulators that may act up- and down-stream to modulate the expression of genes responsive to the exposure to UFP derived from diesel combustion.

### Validation of genes and proteins differentially expressed upon exposure to diesel UFP

The differential expression of a set of DEGs, annotated in the biological processes of *inflammation* and *oxidative stress*, *vasculature developments,* was validated using qPCR (Table 2). The 23 selected targets were manually chosen according to their inclusion in the gene set of *next-maSigPro* cluster and of GSEA hallmarks. Moreover, we proceeded with a deep literature mining analysis referring to specific keywords such as *lung diseases*, *cardiovascular*. Further details are reported in the legend of Table 2. Overall, qPCR results confirmed the expression patterns at 4, 8, 16, 20 h identified by RNA-seq in BEAS-2B exposed to diesel UFP and values from the two types of transcriptional assay resulted highly correlated across all time points (average Pearson correlation value of 0.75 between expression levels from RNA-seq and qPCR; Table 2). Alternatively to qPCR validation, to support the evidences of gene expression profiling and bioinformatics analyses, we quantified the release of four soluble factors, involved in the inflammatory signaling, cell proliferation and regulation of angiogenesis, in cells supernatants. In particular, we evaluated with ELISA assay the protein levels of IL-6, IL-24, EREG, and VEGFA in the protein lysate of BEAS-2B cells exposed to diesel and biomass UFP. As compared to cells exposed to the sole medium, cells exposed to UFP from diesel significantly increased the release of interleukins, IL-6 and MDA-7/IL24, and of EREG and VEGF (Table 3). On the contrary, biomass UFP did not induce any significant modulation of the analyzed proteins (*data not shown*).

**Table 2:** Comparison of qPCR versus RNA-seq of 23 selected DEGs in BEAS-2B cells exposed to diesel UFP. Numbers represent fold changes of separate experiments (*n* = 3) for each time point. Fold changes for both qPCR and RNA-seq are calculated considering exposed cells versus control cells after normalization. Pearson correlation between qPCR and RNA-seq FC for 4, 8, 16 and 20 h resulted in 0.759, 0.772, 0.715 and 0.774 respectively

Gene symbol	qPCR (FC)				RNAseq (FC)				Biological annotations (GSEA^e^)
	4 h	8 h	16 h	20 h	4 h	8 h	16 h	20 h	
*ALDH3A1*	3.49	6.79	6.40	3.74	4.64	4.95	4.65	4.14	Enzyme
*CCL5*	1.78	1.78	−1.04	−1.71	1.85	1.19	1.63	−1.16	Secreted protein (NFK; INF)
*CDKN1A*	1.68	1.51	1.29	1.41	1.56	1.62	1.27	1.34	Transcription factor (NFK; INF)
*CXCL2* ^*a*^	−1.52	−1.04	2.01	3.24	1.28	1.37	1.26	2.09	Secreted protein (NFK; INF)
*EPGN* ^*b*^	6.50	6.04	5.19	4.09	5.92	6.94	8.52	6.06	Secreted protein
*FOSL1*	1.37	1.87	1.94	1.27	1.29	1.75	1.70	1.98	Transcription factor (NFK)
*GREM1* ^*b*^	1.66	2.38	3.50	2.64	1.22	1.97	2.61	2.76	Secreted protein (EMT)
*HES1*	3.56	1.21	1.47	2.34	2.15	−1.19	1.26	1.28	Transcription factor (NFK)
*HMOX1* ^*b*^	3.70	2.95	2.51	2.34	3.46	2.48	1.16	1.35	Enzyme (XEN)
*IER3*	1.88	1.54	2.15	1.63	1.22	1.89	1.85	2.04	Secreted (NFK)
*IGFBP1*	4.20	2.86	3.73	1.95	4.21	4.73	3.82	3.71	Transcription factor (XEN)
*IL1A*	2.47	1.71	2.32	2.08	2.57	1.79	2.25	1.91	Secreted protein (NFK;INF)
*IL1B*	2.10	2.60	3.70	5.30	2.16	1.83	3.73	3.49	Secreted protein (NFK;INF)
*LIF* ^*c*^	1.86	1.72	1.98	1.74	1.36	1.69	1.84	2.25	Secreted protein (NFK)
*MAFF* ^*c*^	1.41	1.41	1.96	1.67	1.52	1.29	1.79	1.45	Transcription factor (NFK)
*NFE2L2*	−1.76	−1.08	1.26	1.94	1.64	1.26	1.28	1.13	Transcription factor (NFK)
*PPP1R15A* ^*d*^	3.39	1.51	−1.12	1.07	2.24	1.88	1.18	1.17	ENZYME (NFK)
*SLC16A6*	3.86	1.58	1.05	2.09	4.09	1.78	1.37	1.29	Solute carrier (NFK)
*SLC7A5*	1.57	2.28	−1.08	2.90	1.25	1.82	1.77	1.98	Solute carrier
*STC2* ^*a*^	3.54	2.25	1.60	1.48	1.94	2.03	1.60	1.53	Secreted factor
*TGIF1*	2.37	1.41	1.15	1.18	1.77	1.23	1.34	1.34	Transcription factor (NFK)
*TNFAIP3* ^*a*^	−1.12	1.22	2.33	2.26	1.01	1.72	1.76	1.48	Transcription factor (NFK;INF)
*TXNRD1* ^*b*^	−1.04	1.92	1.57	2.14	1.43	1.54	1.51	1.55	Enzyme

Gene included in next-maSigPro cluster analysis: ^a^cluster 2; ^b^cluster 9; ^c^cluster 8;^d^cluster 3

^e^GSEA hallmarks gene set legend: NFK *TNF-α signaling* via *NF-kB*, *INF* inflammatory response, *XEN*, xenobiotic metabolism, *EMT*, epithelial mesenchymal transition

**Table 3:** Protein release of BEAS-2B cells exposed to diesel UFP for 20 h assessed by ELISA. Numbers represent mean ± SEM of separate experiments (*n* = 6)

Protein name	Control (pg/ml)	Diesel (pg/ml)	ANOVA test*
IL-6^a^	158 ± 14.7	289 ± 15.7	0.006
IL-24^a^	113 ± 11.6	196 ± 30.9	0.041
EREG	130 ± 2.9	196 ± 4.2	0.032
VEGFA^a^	224 ± 36.2	356 ± 40.7	0.037

*One Way ANOVA (Bonferroni and Dunnett’s), *p* < 0.05 compared to untreated cells

^a^gene temporally modulated in cluster 2 (see Additional file 11: Table S7)

## Discussion

In our study we designed and adopted a time course experiment to investigate, through RNA-seq, the transcriptional changes induced in human bronchial epithelial cells by the exposure to UFP from diesel and biomass combustion. The bioinformatics analysis of transcriptional data reveals that the two different UFP induce, since the earliest time points, different transcriptional dynamics resulting in the activation of specific genes and activation of some common molecular mechanisms, although at very different amplitudes.

The gene expression analysis evidenced that the exposure to diesel UFP induces a stronger modulation of gene transcription as compared to biomass in term of differentially expressed genes, temporal patterns, and pathway activation. We can hypothesized that this difference is connected to the different physicochemical properties of the two types of UFPs. Indeed, in a previous paper [7], we showed that diesel particles are characterized by the presence of PAHs associated with diesel soot, such as pyrene, phenanthrene, benzo[a]anthracene and dibenzo[a,h]anthracene, while biomass particles are enriched in fluoranthene and pyrene. Moreover, we demonstrated that metals are present at higher concentrations in diesel samples as compared to biomass ones. These compounds have been indicated as the main contributors to the effects induced by the combustion particles and differences in their relative content have been suggested to explain the variability of the toxicological responses elicited by such particles [5, 31]. Although a direct association of the different physicochemical properties and the diverse transcriptional effects is still speculative, the previously reported evidences might partly explain the stronger biological response induced by the exposure to diesel UFP.

Bioinformatics analysis allowed the identification of 11 genes significantly induced just 1 h after exposure to diesel UFP and remained constantly over-expressed along the entire time course. This immediate and extended modulation indicates that cell responses are activated shortly after the exposure, and sustained in time. The most modulated genes by diesel UFP were functionally annotated to *xenobiotic metabolism* (*CYP1A1, CYP1B,* and *TIPARP)* and *inflammatory response* (*IL24, EPGN, IL1A,* and *IL1B*). The expression of these genes was also induced by biomass UFP, but at a much lower extent and at later time points (Additional file 6: Table S3). Since PAHs activate the transcription of genes of the xenobiotic metabolism after binding the *AhR* transcription factor [32], these results are concordant with the higher content of PAHs in diesel particles. Several studies reported that *AhR* activation and CYP enzymes increase in in vitro and in vivo models exposed to diesel particles [9, 33], while, to our knowledge, data on biomass and wood particles effects are still lacking. Bonvallot et al. [34] have reported the induction of *CYP1A1* gene expression in 16HBE cells treated with native diesel particles and their organic extracts, but not with stripped particles, showing that the organic compounds are responsible for this over-expression. A more recent study has investigated different diesel samples and found that particles with higher PAHs content were more effective in inducing *CYP1A1* and markers of inflammation and oxidative stress [35]. The same authors analyzed also inflammatory markers, in particular *IL1B*, in monocytes exposed to traffic-derived, wood, and commercial diesel particles, reporting differences in activated signaling pathways in relation to the particle chemical composition [36].

Functional enrichment analysis revealed that both types of particles activate some common molecular mechanisms, although at very different amplitudes. As compared to biomass, exposure to diesel UFP determined higher enrichment scores of biologically relevant gene sets and a larger number of differentially expressed genes in the core enrichment of the modulated pathways. For instance, both diesel and biomass up-regulated genes comprised in the gene sets of *TNF-α signaling* via *NF-kB*, *inflammatory response*, and *hypoxia* but only the exposure to diesel UFP induced this up-regulation along the entire time course. Among the genes belonging to the core enrichments of the TNF-α signaling via NF-kB pathway (Fig. 2) and exerting a profound temporal expression changes by diesel UFP some were confirmed at RNA and protein levels (Tables 2 and 3). By literature mining, we found that these genes of *TNF-α signaling* via *NF-kB* pathway are involved in human diseases such as lung cancer, respiratory, and cardiovascular dysfunctions (Additional file 13: Table S8). Interestingly, *TNF-α* is an inflammatory mediator, which has a tumor-promoting role in various stages of carcinogenesis, induces EMT mediated by *TGF-*β, promotes angiogenesis through the release of *VEGF*, and activates *NF-kB* signaling in models of cancer [37]. Inflammatory mediators as *CCL5*, *CXCL2*, and some *interleukins* (e.g. *IL6, IL8)* contribute to several human diseases through processes including inflammation and angiogenesis [37]. Several studies have shown that exposure to diesel particles and to some diesel compounds (e.g. 1-nitropyrene) triggers the release of such mediators in vitro and in vivo [38–40]. Release of *LIF*, *IL6*, and *IL1B* has been also observed in alveolar and bronchial cells exposed to ambient PM [41]. *IL6* is a well-known marker of diesel and airborne PM exposure, and it has been already suggested to contribute to particles-induced cardiovascular and respiratory diseases [1].. *NFE2L2* (*NRF2*) is an important transcription factor activated by high ROS levels. It regulates genes involved in oxidative stress, inflammation and injury response, and has a protective role in lung and cardiac diseases [42]. In accordance with a recent paper on gene expression modulation after PM exposure [43], our data have shown that diesel exposure regulates many *NFE2L2* target genes involved in glutathione synthesis (*GCLM*), cysteine-glutamate transporter (*SLC7A11*) and antioxidant response (*HMOX1*, *TXNRD1*, and *NQO1*). VEGF (*VEGFA*) is a pro-angiogenic factor with multifunctional roles, including the regulation of both physiological and pathological angiogenesis and vascular remodeling, promoting endothelial cells proliferation and cell migration [44, 45]. It has been reported that an increased expression of VEGF, at the mRNA and protein level, in A549 and BEAS-2B cells, occurs after the exposure to external stimuli such as *IL1B*, *TNF-α* and lipopolysaccharide (LPS) [46]. This event suggests that pulmonary inflammation contributes to the up-regulation and release of VEGF from lung epithelial cells with consequent modulation of lung endothelial cells function and permeability [46]. A recent work from Tseng et al. [47] has reported that endothelial cells exposed to diesel particles release VEGF through the induction of intracellular ROS formation and the secretion of the pro-inflammatory cytokines *TNF-α* and *IL6*. VEGF together with other markers, such as MMPs, HO-1 and ET-1, correlates with the development of atherosclerotic plaque and angiogenesis in mice following exposure to ultrafine PM [48] and diesel particles [49–51]. Noticeably, in diesel we obtained a significant enrichment also for the EMT gene set (Table 1). Since EMT is an important step in the carcinogenic process, this result further supports the tumorigenic potential of exposure to diesel UFP [52]^.^ These observations indicate that diesel particles might contribute to the carcinogenic potential of fine PM [53] at least in cities where traffic contribution to air pollution is relevant.

To extract regulatory connections from the gene expression data, we applied a gene regulatory network analysis and, at least for the exposure to diesel UFP, we discovered some hidden regulators that may act up- and down-stream to modulate the expression of genes responsive to diesel exposure (Fig. 4 and Additional file 15, Figure S5). Among the regulators, we have found the transcription factor *STAT3*, which is activated by pathways involved in inflammation, angiogenesis, and epithelial remodeling [54], and participates in several physiologic cellular function such as proliferation, differentiation, and apoptosis [55, 56]. *STAT3* activation also contributes to pathological processes as oncogenesis, by augmenting tumor invasion and angiogenesis [57, 58]. Cao and coworkers [59] reported the activation of *STAT3* in bronchial epithelial cells exposed to diesel exhaust. *STAT3* target genes include *HES1*, *HMOX1*, *IL6*, *IL24* and *VEGF*, whose modulation has been validated here by qPCR (Table 2) and at protein level (Table 3). The hypoxia inducible-factor-1a *(HIF1a*), another relevant node of the reconstructed gene network, can also induce VEGF transcription, and both these genes were previously found modulated in HUVEC cells exposed to diesel [50]. Consistently with the functional enrichments analyses that highlighted the role of *TNF-α signaling* via *NF-kB*, the gene network pointed out to the transcription factor *NFKB1* (a subunit of the NF-κB complex), previously reported to be activated upon diesel exposure [11]. *NFKB1* has been related to the carcinogenic process through its biological role as cell survival, differentiation, inflammation, and growth [15, 60]. Recently, it has been demonstrated that UFP from vehicular traffic promotes vascular calcification via *NF-κB signaling* activation [61], indicating its possible contribution to cardiovascular diseases. Moreover, the gene network analysis identified, as a relevant node, the oncogene *KRAS*, that was demonstrated to induce the overexpression of EREG [62], a protein that we found to be significantly released by cells exposed to diesel UFP. To our knowledge, EREG modulation has never been reported before in cells exposed to diesel UFP, although we previously reported its release in BEAS-2B and A549 cells exposed to PM [17, 63]. These findings might have a significant role in relation to diesel and PM carcinogenic potency, since deregulation of EREG is known to contribute to the progression of different cancers, including lung, and to lead to an aggressive phenotype and an unfavorable prognosis in KRAS-mutant non-small-cell lung carcinoma (NSCLC) [62, 64]^,^. Overall, the network reconstruction indicated the presence of transcription factors that might activate novel signaling pathways in BEAS-2B cells at diesel exposure.

Nonetheless, the present investigation presents some limitations. BEAS-2B is a cell line commonly used in in vitro models to investigate the potential effect of PM and other airborne pollutants on human bronchial epithelia [65, 66]. However, the use of mono cell culture has clearly disadvantages (e.g. might represent a simplification of the real conditions, lacking the airway multicellular barriers and the air surface liquid secreted by the lung epithelial cells). Recently, systems with cells cultured at Air Liquid Interface (ALI) received considerable attention as an alternative method to expose epithelial cells when investigating the biological effects of ultrafine particle matters [67, 68]. However, studies based on ALI exposure systems still benefit of further experimental validations before able to replace conventional culture condition in air pollution toxicological studies. In the present study, BEAS-2B cells have been exposed to a low dose of UFP (2.5 μg/cm^2^) that, although lower than what reported by Schwarze and co-workers [8], is similar to the deposition dose calculated by Li et al. [69] for the tracheobronchial region. Indeed, starting from an environmental concentration of 79 μg/m^3^/24 h, Li and collaborators indicated that the real life daily exposure might determine a deposition of PM_2.5_ particles up to 200 μg/cm^2^ in the nasopharyngeal tract and up to 2.3 μg/cm^2^ in the tracheobronchial region in persons with healthy conditions. During the winter season, the metropolitan area of Milan might experience environmental concentrations that exceed the level of 79 μg/m^3^/24 h (Regional Environmental Protection Agency ARPA Lombardia) and, thus, the deposition of PM_2.5_ particles in the tracheobronchial region might be higher than the dose adopted here. Nevertheless, transcriptional profiles of cells exposed at this dose indicate that even a low UFP concentration induces a transcriptional modulation. In this respect, we believe that the conditions we selected in our experimental design, although not perfectly mimicking what experienced in a real setting, still constitute an adequate working scenario to recapitulate the impact on human health of UFP from the selected sources of emission.

## Conclusions

Altogether, these data provide new insights into UFP toxicity and shed new light on possible biochemical mechanisms by means of which these particles may act as carcinogens and harmful factors for human cardiovascular disease. In this direction, we think that future investigation should focus on the functional role of genes such as *STAT3*, *HIF1*a, *NFKB*1 and *KRAS* that have emerged as major regulators from the network analysis. An adequate understanding of the mechanisms activated by ambient particles will enable in the future to improve knowledge in the field of PM risk assessment as well as the setting of new strategies towards health protection.

## Additional files

Additional file 1: **Figure S1.** TEM image of diesel particles before (A) and after (B) sonication and resuspension. (PDF 1534 kb)

Additional file 2: **Figure S2.** Alamar Blue viability test of BEAS-2B exposed to increasing concentrations of diesel and biomass UFP. Alamar blue assay (Alamar Blue® Reagent, Catalog nr. DAL1025) was performed according to manufacturers’ instructions. Alamar Blue is a non-toxic dye that changes its colour when active cells metabolize it. Cells viability was proportionally related to the colour of the reagent and it was expressed as percentage (%) of living cells respect to control samples (untreated cells, 100%).The experiments were replicate 3 times and results are expressed as mean percent ± SEM of viable cells in comparison to controls (untreated cells). (PDF 92 kb)

Additional file 3: **Table S1.** Total number of reads and number and percentage of reads mapped by TopHat in each analyzed sample. (XLS 39 kb)

Additional file 4: **Figure S3.** Distributions of normalized cpm counts (in log2) for the 13.309 genes passing the expression filter. The uniform distribution of read counts across all 45 samples supports the elevate quality of sequencing data. Sample IDs are in Additional file 3: Table S1. (PDF 549 kb)

Additional file 5: **Table S2.** FC and *p*-value of the genes differentially expressed after exposure to UFP from diesel combustion (as compared to controls) at the different time points. (XLS 3855 kb)

Additional file 6: **Table S3.** FC and p-value of the genes differentially expressed after exposure to UFP from biomass combustion (as compared to controls) at the different time points. (XLS 3855 kb)

Additional file 7: **Table S4.** Fold change of genes differentially expressed by the exposure to both diesel and biomass UFP. (XLS 67 kb)

Additional file 8: **Figure S4.** Temporal variation of fold changes for the 11 genes that displayed a statistically significant up-regulation along the entire time course of BEAS-2B cells, exposed to diesel UFP. (PDF 232 kb)

Additional file 9: **Table S5.** Functional enrichment of gene sets from GSEA Hallmarks collection in cells exposed to diesel UFP. (XLS 48 kb)

Additional file 10: **Table S6.** Functional enrichment of gene sets from GSEA Hallmarks collection in cells exposed to biomass UFP. (XLS 49 kb)

Additional file 11: **Table S7.** Genes identified by maSigPro as differentially modulated in time during the exposure to biomass or diesel UFP, respectively. (XLS 66 kb)

Additional file 12: **Figure S5.** Expression profiles of genes showing statistically significant expression changes over time in BEAS-2B cells exposed to biomass UFP. Genes have been grouped into 9 clusters showing distinct expression profiles during the time of the experiment. For each plot, the expression values of the clustered genes are represented in either control cell lines (green) or cells after UPF biomass exposure (blue), respectively. Solid line indicates the median instead shadow the median ± S.D. (standard deviation) of the expression values at each time point. (PDF 330 kb)

Additional file 13: **Table S8.** Detail of the filtering criteria for each of the DEGs from network analysis (Fig. 4); for each gene, the number of significant time points, presence in the core enrichment of the selected GSEA hallmarks or the number of Pubmed publications with cardiovascular diseases, lung cancer, or lung diseases terms are indicated. (XLS 78 kb)

Additional file 14: **Table S9.** Biological interactions of genes from network analysis (Fig. 4 and Additional file 15: Figure S6) according to the Reactome FI database; for each interaction, the type of regulation and the score are indicated. Scores range from 0 to 0.99 for predicted while are equal to 1 for manually annotated interactions. (XLS 123 kb)

Additional file 15: **Figure S6.** Complete network from Fig. 4 inclusive of predicted and manually curated interactions according to the Reactome FI database (see Additional file 14: Table S9), respectively as dashed or grey lines. As for Fig. 4, size of DEGs nodes is directly proportional to the number of differentially expressed time points; the FC at each time point in cells exposed to UFP from diesel vs control is also indicated. (PDF 1247 kb)

## Abbreviations

AhR: Aryl hydrocarbon receptor
ARPA: Regional Environmental Protection Agency
DEGs: Differentially expressed genes
EMT: Epithelial mesenchymal transition
FI: Functional Interaction
glm: Generalized linear model
GSEA: Gene Set Enrichment Analysis
INF: Inflammatory response
LPS: Lipopolysaccharide
NES: Normalized enrichment scores
NFK: TNF alfa signaling via NFKB
NSCLC: Non-small-cell lung cancer
PAHs: Polycyclic aromatic hydrocarbons
PM: Particular matter
qPCR: Quantitative Polymerase Chain Reaction
ROS: Reactive oxygen species
TEM: Transmission electron microscopy
UFP: Ultrafine particles
XEN: Xenobiotic metabolism
