# Supplementary figures and images for: Transcriptional profiling of human bronchial epithelial cell BEAS-2B exposed to diesel and biomass ultrafine particles
Source: BMC Genomics. 2018 Apr 27;19:302. doi: 10.1186/s12864-018-4679-9 (PMC5923024; doi:10.1186/s12864-018-4679-9)

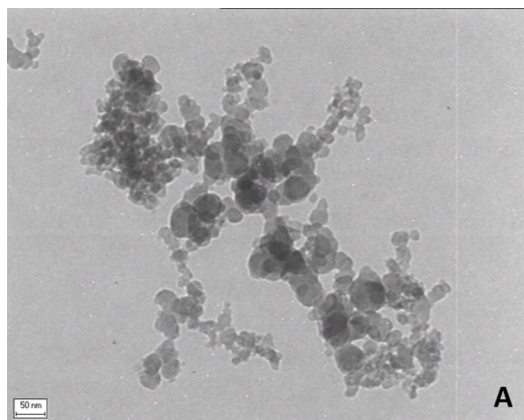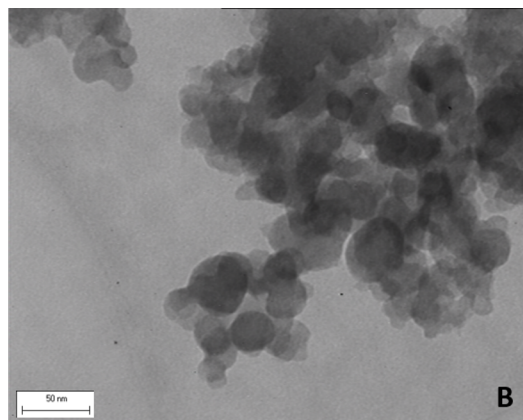

Supplement: Supplementary file 1 — Figure S1. TEM image of diesel particles before (A) and after (B) sonication and resuspension. (PDF 1534 kb) [file 12864_2018_4679_MOESM1_ESM.pdf]

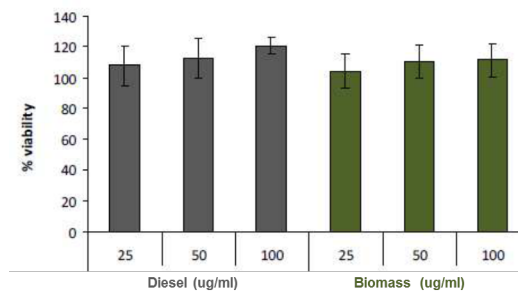

Supplement: Supplementary file 2 — Figure S2. Alamar Blue viability test of BEAS-2B exposed to increasing concentrations of diesel and biomass UFP. Alamar blue assay (Alamar Blue® Reagent, Catalog nr. DAL1025) was performed according to manufacturers’ instructions. Alamar Blue is a non-toxic dye that changes its colour when active cells metabolize it. Cells viability was proportionally related to the colour of the reagent and it was expressed as percentage (%) of living cells respect to control samples (untreated cells, 100%).The experiments were replicate 3 times and results are expressed as mean percent ± SEM of viable cells in comparison to controls (untreated cells). (PDF 92 kb) [file 12864_2018_4679_MOESM2_ESM.pdf]

Normalized read counts (cpm, log<sub>2</sub>)

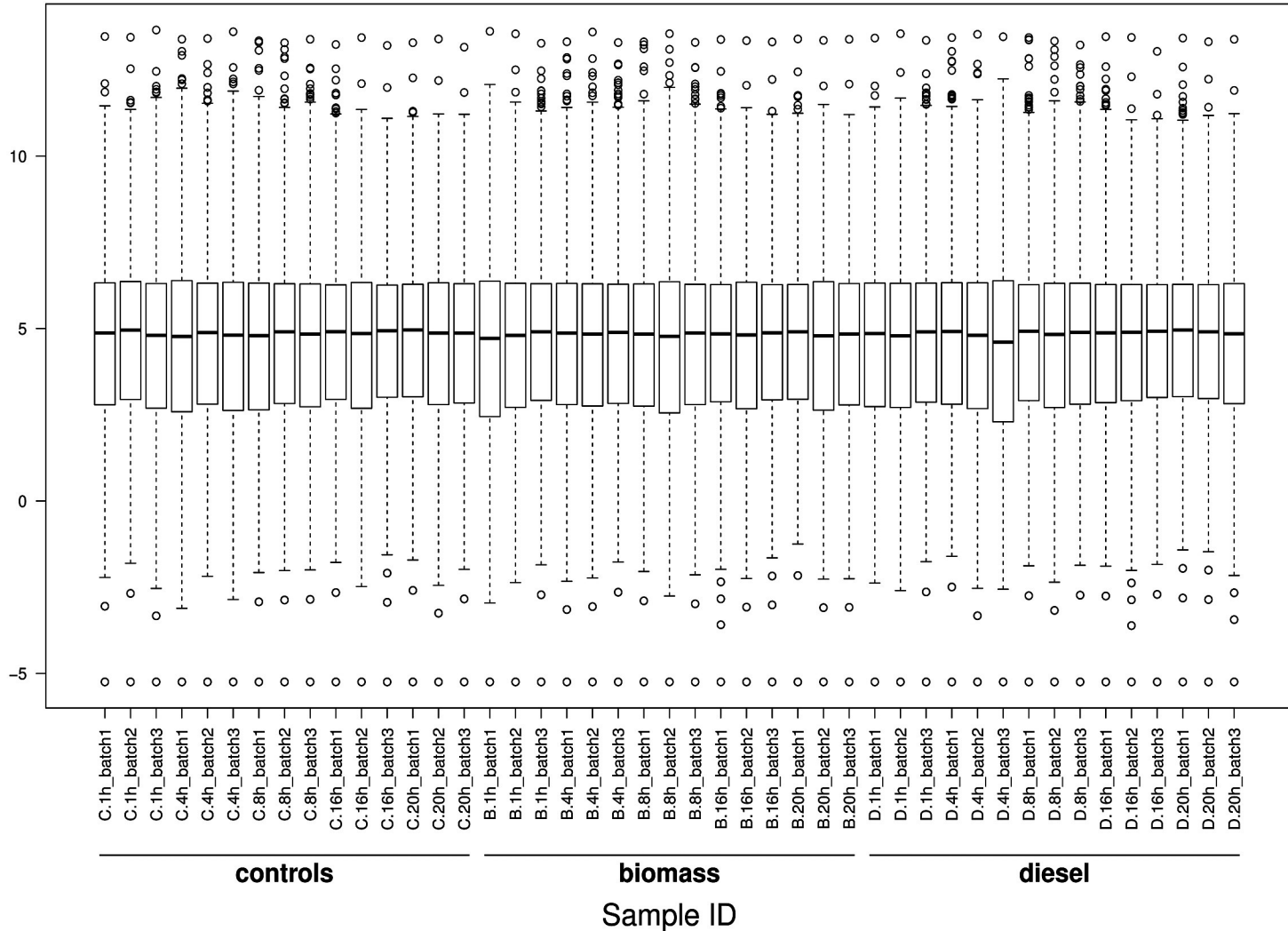

Supplement: Supplementary file 4 — Figure S3. Distributions of normalized cpm counts (in log2) for the 13.309 genes passing the expression filter. The uniform distribution of read counts across all 45 samples supports the elevate quality of sequencing data. Sample IDs are in Additional file 3: Table S1. (PDF 549 kb) [file 12864_2018_4679_MOESM4_ESM.pdf]

**CYP1A1**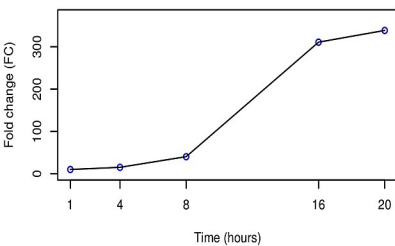**CYP1B1**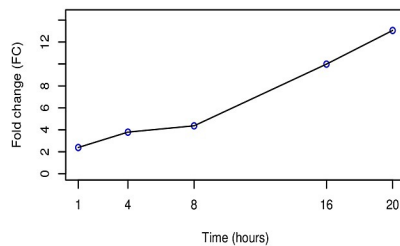**IL24**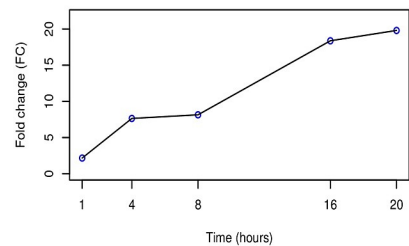**ADAMTS15**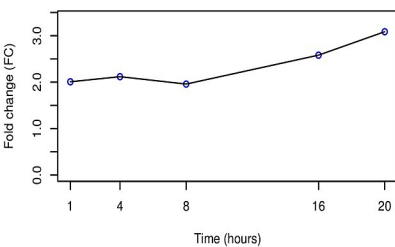**SHISA2**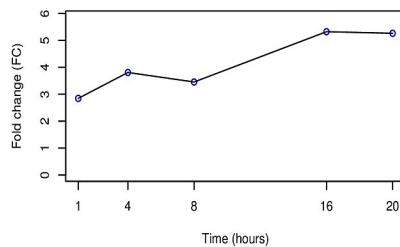**EPGN**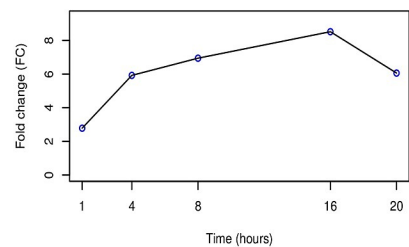**IL1A**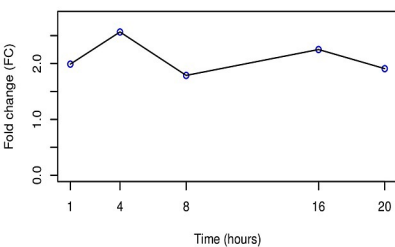**IL1B**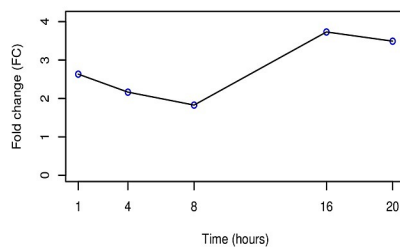**IGFBP1**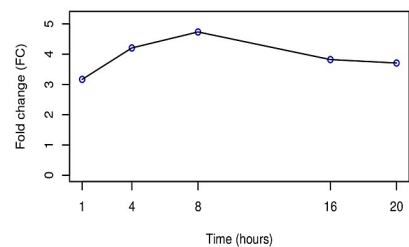**TIPARP**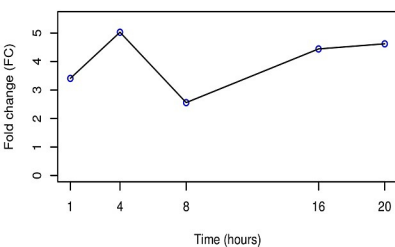**NPTX1**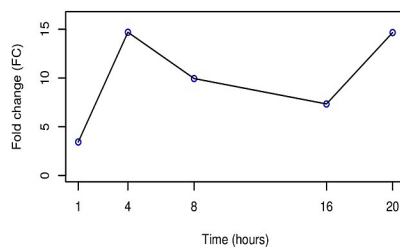

Supplement: Supplementary file 8 — Figure S4. Temporal variation of fold changes for the 11 genes that displayed a statistically significant up-regulation along the entire time course of BEAS-2B cells, exposed to diesel UFP. (PDF 232 kb) [file 12864_2018_4679_MOESM8_ESM.pdf]

**Cluster 1**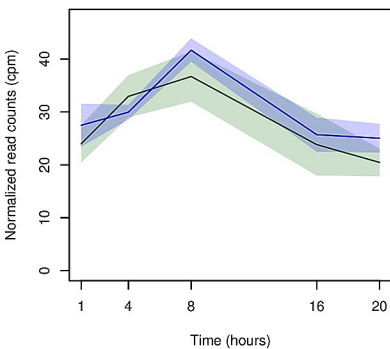**Cluster 2**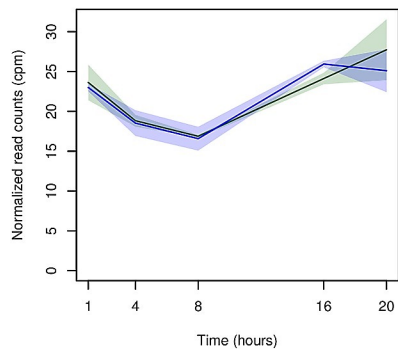**Cluster 3**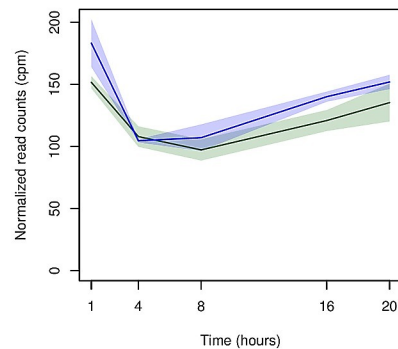**Cluster 4**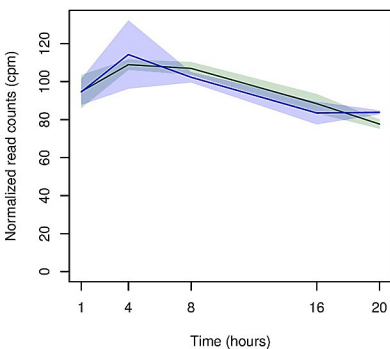**Cluster 5**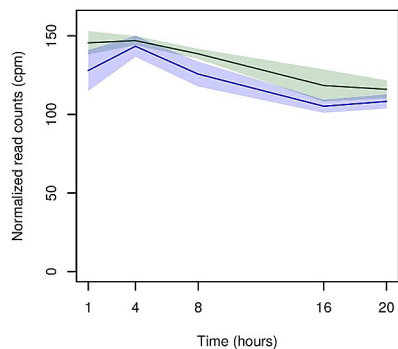**Cluster 6**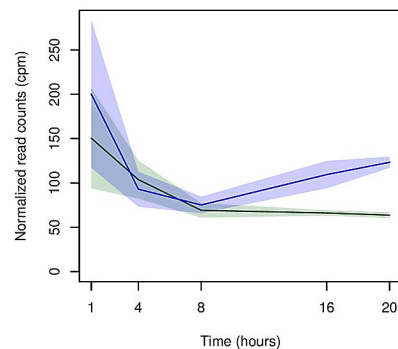**Cluster 7**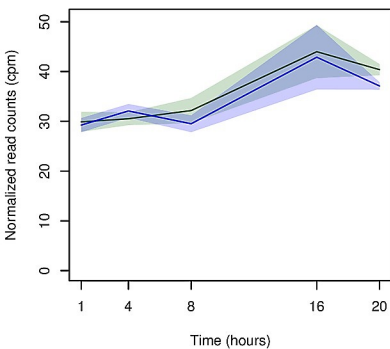**Cluster 8**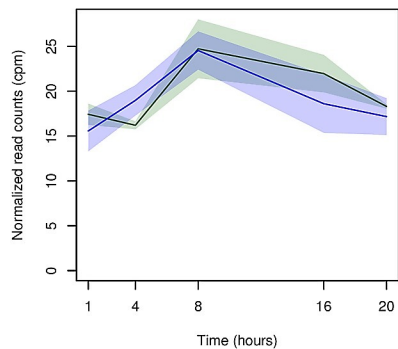**Cluster 9**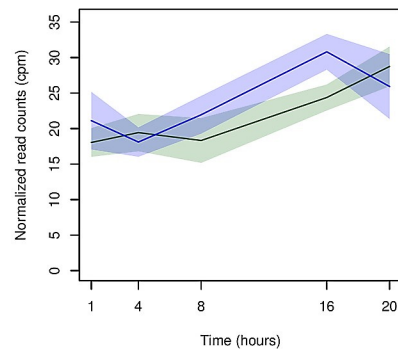

Supplement: Supplementary file 12 — Figure S5. Expression profiles of genes showing statistically significant expression changes over time in BEAS-2B cells exposed to biomass UFP. Genes have been grouped into 9 clusters showing distinct expression profiles during the time of the experiment. For each plot, the expression values of the clustered genes are represented in either control cell lines (green) or cells after UPF biomass exposure (blue), respectively. Solid line indicates the median instead shadow the median ± S.D. (standard deviation) of the expression values at each time point. (PDF 330 kb) [file 12864_2018_4679_MOESM12_ESM.pdf]

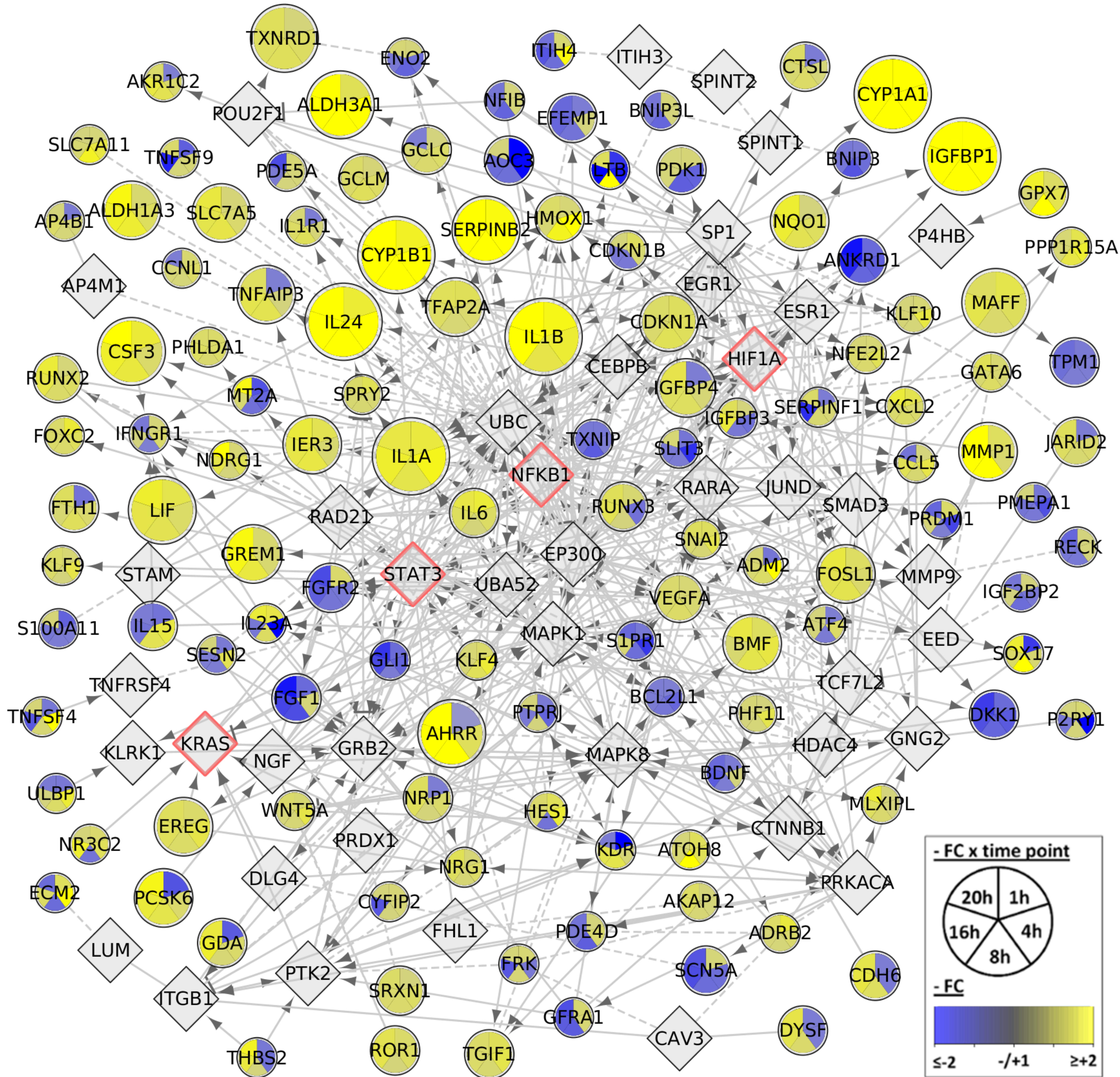

Supplement: Supplementary file 15 — Figure S6. Complete network from Fig. 4 inclusive of predicted and manually curated interactions according to the Reactome FI database (see Additional file 14: Table S9), respectively as dashed or grey lines. As for Fig. 4, size of DEGs nodes is directly proportional to the number of differentially expressed time points; the FC at each time point in cells exposed to UFP from diesel vs control is also indicated. (PDF 1247 kb) [file 12864_2018_4679_MOESM15_ESM.pdf]
